# Association of Dietary Patterns with Weight Status and Metabolic Risk Factors among Children and Adolescents

**DOI:** 10.3390/nu13041153

**Published:** 2021-03-31

**Authors:** Seulki Oh, So Yeong Lee, Do-Yeon Kim, Sarah Woo, YoonMyung Kim, Hye-Ja Lee, Han Byul Jang, Sang Ick Park, Kyung Hee Park, Hyunjung Lim

**Affiliations:** 1 Department of Medical Nutrition, Graduate School of East-West Medical Science, Kyung Hee University, Yong-in 17104, Korea; 90selki@gmail.com (S.O.); zxc931102@khu.ac.kr (S.Y.L.); 2 Research Institute of Medical Nutrition, Kyung Hee University, Seoul 02447, Korea; rou_@naver.com; 3 Department of Family Medicine, Hanllym University Sacred Heart Hospital, Anyang 14068, Korea; hjejcross@naver.com; 4 University College, Yonsei University International Campus, Incheon 21983, Korea; yoonkim@yonsei.ac.kr; 5 Center for Biomedical Sciences, Korea National Institute of Health, Cheongju, Chungbuk 28159, Korea; hyejalee@korea.kr (H.-J.L.); greatstar@korea.kr (H.B.J.); parksi@nih.go.kr (S.I.P.)

**Keywords:** dietary patterns, obesity, children, adolescents, factor analysis, South Korea

## Abstract

Unhealthy dietary patterns are associated with obesity in children and adolescents. However, few studies have investigated the relationships between dietary patterns and obesity-related metabolic disorders among Asians. We identified dietary patterns in children and adolescents and examined the associations between these patterns and obesity, insulin resistance, and metabolic syndrome in South Korea. This study is a cross-sectional design. We used baseline data from an intervention study of 435 Korean children and adolescents aged 6–17 years. Insulin resistance was assessed as HOMA-IR ≥ 2.6. Metabolic syndrome was diagnosed by cardiovascular disease risk factor clustering. Dietary intakes were estimated using 3-day food records. Factor analysis was used to obtain dietary patterns, and we examined the associations between dietary patterns and obesity-related markers adjusted for potential covariates. Three dietary patterns were identified as fast food and soda (FFS), white rice and kimchi (WRK), and oil and seasoned vegetable (OSV) patterns. Compared with participants in the lower intake of FFS pattern, those in the top intake were associated with a higher waist circumference (WC) (β = 1.55), insulin level (β = 1.25), and body mass index (BMI) (β = 0.53) and it was positively associated with HOMA-IR ≥ 2.6 (OR = 2.11; 95% CI: 1.227–3.638) (*p* < 0.05). WRK pattern was associated with lower weight and higher HDL cholesterol, and the OSV pattern was associated with a lower weight, WC, and insulin level (*p* < 0.05). The FFS pattern showed a positive relation with WC, serum insulin, and BMI, and the other two dietary patterns indicated a preventive effect of those parameters. The FFS pattern was associated with significantly elevated insulin resistance among children and adolescents.

## 1. Introduction

Childhood obesity is one of the serious public health challenges worldwide [1]. In Korea, the prevalence of obesity in children and adolescents increased 1.5 times from 11.2% in 2007 to 17.3% in 2017, and severe obesity increased 1.6 times during the same period [2]. Overweight and obese children and adolescents are likely to maintain their status into adulthood and are at higher risks for developing chronic diseases such as type 2 diabetes and cardiovascular disease [3]. 

The causes of childhood obesity are multifactorial, including genetic, behavioral, and environmental factors [4]. Among these, dietary factors are the key cause of overweight and obesity [5]. Previous studies indicated that intake of fast food is associated with obesity [6], and frequent snacking is associated with a high risk of abdominal obesity in children and adolescents [7]. From a single-nutrient/food approach, a high intake of saturated fat and carbohydrate (e.g., sugars, soft drinks, instant noodles, and fast foods) increases the risk of obesity in children and adolescents [8]. However, focusing on a single nutrient or food may not explain the association between eating habits and obesity risk in childhood [9]. This is why dietary pattern analysis, which examines dietary patterns rather than a single nutrient or food intake, has been used to investigate associations between food intake and diseases in certain population segments [9]. 

Although there have been several studies analyzing the dietary patterns of children and adolescents, most of the studies have been conducted in Western countries, e.g., Europe and Australia, and it is known that they mainly show a relationship in the Westernized dietary pattern with obesity [10,11] and also the risk of metabolic syndrome [12]. Overweight and obese children and adolescents tend to eat a Western dietary pattern, which is composed of bread, pizza, and burgers [13], and this pattern is associated with increased risk of non-communicable diseases such as obesity in Iranian adolescents aged 12–19 years [14] and cardiometabolic risk in Australian adolescents [12]. Reversely, the dietary pattern which is rich in vegetables and fruits has been associated with a lower prevalence of obesity [14]. However, there is a lack of research concerning the associations between dietary patterns and obesity-related metabolic conditions in children and adolescents, especially in Asian countries. To fill the literature gap, we identified dietary patterns among children and adolescents in South Korea, and examined the associations between dietary patterns and obesity, insulin resistance, and metabolic syndrome.

## 2. Materials and Methods

### 2.1. Study Design and Subjects

This cross-sectional study used the baseline data from a prior study that is the Intervention for Children and Adolescent obesity via Activity and Nutrition (ICAAN) for our analysis. The ICAAN study was designed to examine the effects of multidimensional intervention including circuit training and balanced diet in children and adolescents in Korea [15]. A total of 435 Korean children and adolescents aged 6–12 years (n = 336) and aged 13–17 years (n = 99) were recruited for this study, respectively. 

We included those who participated in the ICAAN study who met the criteria for normal weight children and adolescents (body mass index (BMI) 5–85th percentile of age and sex-specific BMI), overweight and obese children and adolescents (BMI 85th-120% of the 95th percentile), and severely obese participants (BMI ≥ 120% of the sex- and age-specific 95th percentile BMI) according to the 2017 Korean National Growth Chart [16,17], and excluded 53 subjects, whose anthropometric, biochemical, and dietary intake data were missing. A total of 435 Korean boys (n = 261) and girls (n = 174) were included in the dietary pattern study. This study was approved by the Institutional Review Board of Hallym University Sacred Heart Hospital (approval No. 2016-I135).

### 2.2. Anthropometric Measurements and Blood Pressure

Body weight (Wt), height (Ht), waist circumference (WC), hip circumference (HC), and systolic and diastolic blood pressure (SBP and DBP) were measured by a trained staff. Ht and Wt were measured to the nearest 0.1 cm and 0.1 kg, respectively (DS-103, Dongsan Jenix Co., Seoul, South Korea). Body mass index (BMI) was calculated based on Wt and Ht (kg/m^2^). In addition, BMI z-score was calculated by BMI-for-age percentiles together with the Lambda-Mu-Sigma (LMS) method of Cole and Green [18], which provides a way of obtaining normalized growth percentile standards, based on 2017 Korean National Growth Charts for Children and Adolescents [16]. WC was measured with participants standing straight using a flexible tapeline at the midpoint of the lower rib and the iliac crest to the nearest 0.1 cm. HC was measured using a flexible tapeline at the horizontal circumference of the highest point of the buttocks to the nearest 0.1 cm. The waist–hip ratio (WHR) was calculated based on recorded WC and HC measurements. Both SBP and DBP were measured two times in the right arms of participants in a seated position following a rest of at least 5 min by an automatic sphygmomanometer (HEM-907, OMRON Healthcare Co., Kyoto, Japan) and the average value was used for analysis.

### 2.3. Biochemical Assessments

After participants fasted for 8 to 12 h, venous blood samples were collected from peripheral blood vessels. Obtained blood samples were transferred to the Seegene Medical Foundation (Seoul, South Korea) for analysis. Triglyceride (TG, mg/dL) level was measured in an enzymatic assay (Cobas 8000 C702, Roche, Mannheim, Germany). Level of high-density lipoprotein cholesterol (HDL-C, mg/dL) and low-density lipoprotein cholesterol (LDL-C, mg/dL) were also measured by homogeneous enzymatic colorimetric tests (Cobas 8000 C702, Roche, Mannheim, Germany). Fasting blood glucose (FBG, mg/dL) was measured using an ultraviolet assay with the hexokinase method (Cobas 8000 C702, Roche, Mannheim, Germany). Fasting serum insulin level (μU/mL) was measured by electrochemiluminescence immunoassay (Cobas 8000 E802, Roche, Mannheim, Germany). Homeostatic model assessment for insulin resistance (HOMA-IR) was calculated using the following formula: (Fasting serum insulin (μU/mL) × Fasting blood glucose (mg/dL))/405. 

### 2.4. Criteria of Metabolic Risk Factors

The insulin resistance is determined using the homeostatic model assessment insulin resistance (HOMA-IR). The HOMA-IR criteria for children and adolescents determined the insulin resistance criterion (HOMA-IR ≥ 2.6) using a range that increases the risk of cardiovascular risk [19] and it was calculated by insulin and fasting blood glucose. The metabolic syndrome is defined by criteria from cardiovascular disease risk factor clustering (CVD-RFC) in children and adolescents [20,21,22]. According to the CVD-RFC, metabolic syndrome in children and adolescents is present if two or more of the following factors must be met. Abdominal obesity was defined as WC (cm) ≥ 90th percentile value of age and sex according to the 2007 Korean National Growth Charts, TG ≥ 110 mg/dL, HDL-C < 40 mg/dL, SBP or DBP (mmHg) ≥ 90th percentile value of age and sex according to the 2007 Korean National Growth Charts, FBG ≥ 100 mg/dL. 

### 2.5. Dietary Assessments

Dietary intake was estimated using a 3-day food record [23] which is written by each participant and their caregiver. Then, we confirmed the food record through their parents or caregiver. Participants were asked to record their intake of meals and snacks, including beverages during a nonconsecutive period of three days (two weekdays and one weekend day). They also were asked to record ingredients of meals as well as portion sizes. A well-trained dietitian reviewed and confirmed the written 3-day food record using food models during face-to-face interviews to increase precision in reporting. Research-based typical food intake data were used to calculate averages and energy, macronutrients, and micronutrients were analyzed using a computer-aided nutritional analysis program (CAN-Pro) web version 5.0 (Korean Nutrition Society, 2016).

### 2.6. Dietary Patterns Analysis

All food items were classified into 25 food groups, based on the generalized 17 food groups in the Korean Nutrient Database of the Korean Nutrition Society. This study separated the original grains group into 6 subgroups to indicate representative food items that comprise typical consumption in Korean children and adolescents. Among the food groups, the meat and fish groups were combined into a single food group because they are the most consumed animal protein sources in South Korea [24]. The list of food groups is presented in Supplemental Table S1. We conducted factor analysis (based on principal component analysis (PCA)) to investigate dietary patterns based on the percentages of total energy intake in the 25 food groups. Factor analysis using SAS proc factor (SAS Institute Inc.) was rotated via an orthogonal transformation (varimax rotation) to obtain a simplistic structure and distinct interpretation. To determine the number of dietary patterns, we considered eigenvalues greater than 1.4 and scree plots. Also, to describe the dietary patterns, food groups were retained in a factor if they had a correlation of 0.25 with that factor. The dietary pattern was named based on prominent food items. We divided Factor scores into three tertiles on the basis of their contribution to each dietary pattern, and an increase from T1 to T3 was assumed.

### 2.7. Statistical Analysis

All analyses were performed using SAS software (SAS 9.4; SAS Institute Inc., Cary, NC, USA). The characteristics of participants were compared by Student *t*-test and Chi-square test. Each dietary pattern was subdivided into tertiles. The tertiles were compared using a generalized linear model adjusted for age, sex, total energy intake, house income and parental education level, followed by Tukey’s post hoc tests. We assessed the associations between dietary patterns and obesity-related markers (Wt, WC, BMI z-scores, insulin, TG, LDL-C, and HDL-C) using linear regression. Subsequently, a multivariable logistic regression model was used to estimate the odds of obesity, insulin resistance, and metabolic syndrome for each dietary pattern. This study also adjusted for potential covariates including age, sex, and energy intakes. The variables were described as odds ratio (OR) and 95% confidence intervals (95% CI). Following regression analysis, variable median scores by tertiles of dietary patterns were used to examine trends. All statistical significance was defined at *p* < 0.05.

## 3. Results

General characteristics of study participants are presented in Table 1. In comparing the boys and girls, the dietary intakes of energy (boys = 2289.2 kcal/day and girls = 2014.0 kcal/day), carbohydrate (boys = 303.5 g and girls = 270.3 g), protein (boys = 88.4 g and girls = 78.6 g), and dietary fat (boys = 77.8 g and girls = 66.7 g) were significantly higher in boys. Additionally, boys consumed higher amounts of MUFA (monounsaturated fatty acid, boys = 7.9 g) than girls (girls = 6.1 g; *p* < 0.047). Wt (64.7 kg), WC (87.2 cm), WHR (0.91), SBP (119.2 mmHg), and BMI z-score (2.3) were significantly higher in boys than those of girls (Wt (58.4 kg), WC (79.2 cm), WHR (0.85), SBP (113.5 mmHg), and BMI z-score (1.9); *p* < 0.01). However, biochemical markers, e.g., TG, FBG, insulin, HOMA-IR and socioeconomic status (SES), e.g., household income, paternal education were not significantly different between boys and girls. Each prevalence of metabolic syndrome, insulin resistance and obesity (BMI z-score ≥ 2) is 61.0%, 71.7%, and 68.7% respectively.

Factor analysis revealed three dietary patterns, and they are presented in Table 2. The three patterns were named based on food items. The fast food and soda (FFS) pattern was characterized by high intakes of fast food, carbonated beverages, and instant ramen, and low consumption of soups, vegetables and mushrooms, fruit, and milk and dairy products. The white rice and kimchi (WRK) pattern was characterized by high intakes of white rice, Kimchi, processed food, and eggs, and low intakes of snack and cereals, flour and rice cakes, noodles, and sugars. This represents simplified Korean food. In addition, the oil and seasoned vegetable (OSV) pattern represents a high consumption of seasoned vegetable or vegetable stir-fry, characterized by high intakes of vegetable oil, sugars, seasonings, potatoes, nuts, and vegetable and mushrooms. These patterns accounted for 1.99% (FFS), 1.99% (WRK) and, 1.69% (OSV) of total variance explained in food intakes, respectively (data not shown). 

The difference of intakes, anthropometric and biochemical markers according to tertile of dietary patterns is summarized in Table 3. After adjusting for age, sex and energy intake, participants with higher FFS pattern had greater energy intake. Energy and fat intakes gradually increased, whereas carbohydrate intake decreased with higher tertile of FFS pattern (*p* < 0.000). In contrast, higher tertile of the WRK pattern showed less energy intake and lower fat intake, whereas carbohydrate intake increased with higher tertile of the WRK pattern (*p* < 0.000). Similarly, the intake of carbohydrates was increased, and fat was decreased in the top tertile of the OSV pattern (*p* < 0.000). For anthropometric characteristics, Wt, WHR, and, BMI z-score gradually increased with higher tertile of FFS pattern (*p* < 0.000). On the other hand, Wt gradually decreased, but DBP increased in higher tertiles of WRK pattern (*p* < 0.000). Additionally, Wt, DBP, WHR, and BMI z-score gradually decreased with higher tertile of the OSV pattern (*p* < 0.000). In terms of comparing biochemical characteristics across tertiles of the dietary patterns, the higher tertiles of the FFS pattern showed increased levels of insulin, HOMA-IR, and triglyceride, whereas HDL-C gradually declined (*p* < 0.000). Conversely, insulin level decreased in higher tertiles of the WRK pattern (*p* < 0.000). Subjects following the OSV pattern showed decreased levels of insulin, HOMA-IR and triglyceride in higher tertiles (*p* < 0.000).

Association between tertiles of discrete dietary pattern and obesity and metabolic risk markers are shown in Table 4. After adjusting for age, sex, and energy intake, the FFS pattern had positive association with WC, SBP, insulin, weight, BMI z-score and HOMA-IR (β = 1.55, 1.31, 1.25, 1.00, 0.16, and 0.34, respectively; *p* < 0.05), but the pattern was negatively associated with HDL-C (β = −0.03, *p* < 0.05). The WRK pattern had association with WC, insulin, and HDL-C (β = 0.44, −0.16 and 0.58, respectively; *p* < 0.05). Similarly, the OSV pattern had reverse association with Wt, SBP, WC, BMI z score, insulin and HOMA-IR (β = −1.57, −0.76, −0.86, −0.10, −1.27 and −0.30, respectively; *p* < 0.001). However, the OSV pattern was positively associated with HDL-C (β = 0.17, respectively; *p* < 0.05).

The association between obesity, insulin resistance, and metabolic syndrome across tertile of dietary patterns is shown in Table 5. After adjustment, the FFS pattern had twofold higher odds of insulin resistance (OR = 2.11; 95% CI: 1.23–3.64) compared with those in the T1. Participants with the highest intake of FFS pattern had a 1.7 times higher odds of BMI z-score ≥ 2 (OR = 1.67; 95% CI: 1.01–2.76), and nearly 1.8 times more likely to have prevalent metabolic syndrome (OR = 1.79; 95% CI: 1.11–2.87) compared with those in the T1. In the model I (crude), however, the association was not significant after adjustment of age, sex, energy intake, household income, and paternal education level (model II). 

## 4. Discussion

This study identified three major dietary patterns among Korean children and adolescents; FFS, WRK, and OSV patterns. After adjusting for covariates, the FFS pattern was positively associated with Wt, SBP, WC, insulin, and HOMA-IR, and caused a significantly higher prevalence of insulin resistance. The WRK pattern was associated with lower Wt and insulin and associated with higher WC and HDL-C. The OSV pattern was associated with lower Wt, WC, insulin, and HOMA-IR, and associated with higher HDL-C. 

To date, the majority of published studies on dietary patterns have reported that dietary patterns of children and adolescents in Asia had been increasingly Westernized [25,26]. Korea is not an exception. The secular trends of dietary pattern revealed that the traditional Korean patterns (higher consumptions of white rice, kimchi, vegetables, and fish) have declined, while a Western pattern has increased in children and adolescents aged 10–19 years [27]. According to related previous studies in the United States and European countries, dietary patterns described as high fat intake and high calories increase the risk of obesity compared to dietary patterns of whole grains, low-fat dairy products, vegetables, and fruits [10,28]. However, in a Chinese study, the modern dietary pattern (characterized by high intake of milk, fast foods, eggs, meat, poultry, and cake) has a higher relationship with obesity than traditional Chinese dietary patterns (represented by high consumption of vegetables, rice, pork, and legumes) [25]. This is similar to the results in Asian countries such as Japan [26]. According to the results using Korean national data, Korean traditional meal patterns, Western patterns, and healthy patterns are derived [27]. However, in our study, the proportion of obese children is high, which stem from different patterns from that of the previous Korean dietary patterns study [27]. 

Consistent with previous research [13,29], our study found a similar dietary pattern designated as FFS pattern which is composed of fast food, carbonated beverages, and instant ramen. According to a previous study about dietary pattern for adolescents, it was found that high consumption of fast food and red meat has association with increasing overweight in Lebanese adolescents aged between 13 and 19 years [13]. Additionally, previous studies have confirmed that high consumption of pizza, burgers, sugar drinks is associated with increasing high BP [14] and increasing the risk of insulin resistance [30]. It was reported to be associated with not only a high intake of energy, sodium, SFA, and sugar, but also a low intake of fiber and PUFA [14,30]. In the present study, high consumption of the FFS pattern did not increase the intake of sodium and sugar (data are not shown), but the results concerning energy, fiber, and PUFA intake were similar to the previous [27,31]. In addition, participants with higher fast food and soda consumption had a higher prevalence of insulin resistance and higher prevalence of the metabolic syndrome, but it was not significant after adjustment that is due to a lack of statistical power. The earlier study confirmed that the incidence of metabolic syndrome is rising commensurately with the rising intake of fast food and soda in certain populations [6]. Because the high-carbohydrate foods contribute to insulin resistance, it prevents glucose from being used as an energy source, increases the synthesis of TG and affects the concentration of lipid and lipoprotein, which causes metabolic disorders to occur simultaneously [32]. Although intake of the FFS pattern in this study does not increase carbohydrate intake, the risk of metabolic syndrome could be increased as it is related to increased fat intake and associated with increased risk of insulin resistance. Also, one other study said that even though fast food is known to be unhealthy, unhealthy dietary patterns including fast food have a stronger correlation with metabolic disorders than fast food consumption on its own [33]. Therefore, we should carefully consider whether eating the FFS pattern might contribute to metabolic syndrome.

A WRK dietary pattern composed of white rice, kimchi, egg, and processed food in Korean children and adolescents might be influenced by modernization [26]. However, a previous study reported that subjects whose weight is close to normal weight are likely to have a Korean style dietary pattern [34], and another study in Korean adults identified that the traditional dietary pattern has a negative association with central obesity and association with decreased risk of metabolic syndrome [35]. Consistent with those results, this study found that the WRK pattern has a negative association with Wt, BMI z-score and insulin level in our subjects. However, it was not shown to be associated with a lower prevalence of metabolic syndrome or obesity. This may be because, while traditional dietary patterns had low-fat content and a high portion of vegetables (established contributors to good health) [35], the WRK pattern in this study did not include the vegetable and instead contained processed food. Especially, adolescents are experiencing a dramatic increase in intake of Westernized food including processed food among adolescents aged 12–14 years in Seoul, Korea [36]. Therefore, further study is necessary to monitor children and adolescents’ dietary patterns in the long term to examine periodically their dietary patterns in Korea.

The OSV pattern which is characterized by the vegetable oil, sugars, seasonings, potatoes, nuts and vegetable and mushrooms pattern had a negative association with Wt, WC, BMI z-score and insulin level. Vegetable intake was inversely associated with obesity in children and adolescents in China [31], vegetable and vegetable oils were associated with lower insulin resistance in children and adults [37,38]. Also, the nut consumption had benefits for adolescents that were inversely associated with BMI z-score and linked to reduced glucose and lipid risk for cardiovascular disorders [39]. The benefits from the OSV pattern originate from eating a variety of vegetables and nuts [36,39]. It is because the unsaturated fatty acids which are rich in vegetable oil are known to be inversely associated with WC, BP, and insulin resistance. In addition, dietary fiber which is rich in nuts and vegetables is well known to be related to help the improvement of BP, insulin resistance, and obesity in adult women and obese children and adolescents [37,38,40]. Consistent with other studies, the oil and seasoned vegetable pattern had a high intake of fiber and unsaturated fatty acids [41] (data not shown). The OSV pattern, which includes such nutritional factors, is believed to reduce the risk of cardiometabolic disease factors such as lower Wt, WC, BMI z-score and insulin level and increasing HDL-C. However, according to a previous study, high consumption of vegetable oil may increase energy density [42]. Therefore, it is necessary to consider excessive consumption of vegetable oil.

Additionally, the OSV pattern in this study also included sugars and seasonings such as salt and soy sauce. Accordingly, it is difficult to conclude definitively that this particular OSV pattern is beneficial for childhood because consumption of a salt-rich dietary pattern should increase the risk of high blood pressure [14] and higher sugar intake increases fasting glucose and insulin resistance [43]. Therefore, eating with high consumption of vegetable oil (such as nuts, sesame oil, and perilla oil, etc.) and vegetables are believed to be beneficial to the cardiometabolic disease factors. 

The present study has several strengths. This is the first study to examine the association of dietary patterns and cardiometabolic disease factors in children and adolescents, including children and adolescents with severe obesity in Korea. Then, we used a 3-day food record and confirmed it by the participants’ caregivers and a well-trained clinical dietitian. Moreover, we used the SES variables for analysis (e.g., Monthly family income, Parental education).

Despite these strengths, several limitations may have affected this study. Since this study was a cross-sectional design, it is not possible to retrospectively evaluate whether dietary patterns are causal with insulin resistance, metabolic syndrome, and other indicators. Second, there was a possible under-reporting of dietary intake that may occur in obese and severely obese participants. Third, even though physical activity is one of the major factors in obesity, we were unable to use physical activity as a variable due to lots of missing data of physical activity on the participants. At least, this study should be interpreted assuming that there are no significant differences in the level of physical activity of the subjects. 

## 5. Conclusions

We found three dietary patterns of the FFS, WRK, and OSV patterns in Korean children and adolescents. Among these dietary patterns, the high intake of the FFS pattern was associated with an increased risk of insulin resistance. These findings will be of some help to counseling and education in preventing obesity in childhood and adolescence. However, further studies are needed to investigate more objectively the causal relationship between dietary patterns and cardiometabolic risk using prospective data.

## Figures and Tables

**Table 1:** General characteristics of Korean children and adolescents by gender.

Variables	Total (*n* = 435)			Boys (*n* = 261)			Girls (*n* = 174)			*p*-Value *
**Age (years)**	11.11	±	1.98	10.92	±	1.82	11.39	±	2.16	0.017
**Dietary intake**										
Energy (kcal)	2179.11	±	565.45	2289.20	±	585.90	2014.00	±	−490.50	<0.0001
Carbohydrate (g)	290.19	±	77.26	303.50	±	79.24	270.30	±	69.80	<0.0001
Protein (g)	84.52	±	25.84	88.44	±	26.79	78.64	±	23.21	<0.0001
Fat (g)	73.34	±	27.35	77.78	±	28.91	66.68	±	23.38	<0.0001
C:P:F ratio (%)	53.8:15.7:30.6			53.5:15.6:30.9			54.2:15.8:30.1			
Cholesterol (mg)	182.73	±	29.17	182.30	±	28.37	183.40	±	30.41	0.686
SFA (g)	7.44	±	5.86	7.87	±	5.54	6.80	±	6.27	0.062
MUFA (g)	6.80	±	6.25	7.28	±	5.92	6.07	±	6.66	0.047
PUFA (g)	4.94	±	4.51	5.26	±	4.33	4.46	±	4.74	0.069
**Anthropometrics**										
Height (cm)	151.73	±	11.10	152.70	±	11.40	150.30	±	10.50	0.026
Weight (kg)	62.16	±	19.96	64.66	±	19.85	58.41	±	19.59	0.001
WC (cm)	84.02	±	15.22	87.23	±	14.27	79.19	±	15.37	<0.0001
HC (cm)	94.06	±	13.09	95.14	±	11.68	92.45	±	14.85	0.045
WHR	0.89	±	0.09	0.91	±	0.07	0.85	±	0.10	<0.0001
SBP (mmHg)	116.92	±	17.16	119.20	±	16.55	113.50	±	17.54	0.001
DBP (mmHg)	67.58	±	10.17	68.37	±	9.77	66.39	±	10.66	0.046
BMI (kg/m^2^)	26.39	±	5.73	27.13	±	5.38	25.28	±	6.07	0.001
BMI z-score	2.17	±	1.57	2.34	±	1.43	1.92	±	1.74	0.007
**Household income (*n,* %) ^(1)^**										
Low income	123		(28.28)	73		(27.97)	50		(28.74)	0.983
Middle income	113		(25.98)	69		(26.44)	44		(25.29)	
High income	87		(20.00)	51		(19.54)	36		(20.69)	
**Paternal Education level (*n*, %)**										
≤Middle school graduation	8		(1.84)	5		(1.92)	3		(1.72)	0.725
Graduated high school	132		(30.34)	84		(32.18)	48		(27.59)	
>High school graduation	241		(55.40)	139		(53.26)	102		(58.62)	
**Cardiometabolic risk (*m*, %) ^(2)^**										
Obesity	299		(68.70)	183		(70.11)	96		(55.17)	0.164
Insulin resistance	312		(71.70)	192		(73.56)	120		(68.97)	0.297
Metabolic Syndrome	268		(61.60)	163		(62.45)	105		(60.34)	0.658
**Biochemistry**										
Triglycerides (mg/dL)	105.33	±	52.39	103.00	±	50.09	108.80	±	55.63	0.255
Total cholesterol (mg/dL)	182.73	±	29.17	182.30	±	28.37	183.40	±	30.41	0.686
HDL cholesterol (mg/dL)	52.11	±	12.53	51.79	±	12.15	52.58	±	13.11	0.522
LDL cholesterol (mg/dL)	109.55	±	25.06	109.90	±	24.30	109.10	±	26.23	0.745
Fasting glucose (mg/dL)	89.47	±	8.76	89.99	±	8.82	88.70	±	8.64	0.133
Insulin (uU/mL)	19.48	±	13.69	20.06	±	14.46	18.61	±	12.43	0.265
HOMA-IR	4.32	±	3.38	4.51	±	3.73	4.05	±	2.77	0.138

Abbreviation: C:P:F ratio, ratio of carbohydrate to protein to fat; SFA, saturated fatty acid; MUFA, monounsaturated fatty acid; PUFA, polyunsaturated fatty acid; WC, waist circumference; HC, hip circumference; WHR, waist–hip ratio; SBP, systolic blood pressure; DBP, diastolic blood pressure; BMI, body mass index; HOMA-IR, homeostatic model assessment for insulin resistance. * *p*-Values for difference between sex from Chi-squared test (categorical variables) or student *t*-test (continuous variables). Values are Mean ± SD. ^(1)^ Low income: ≤4,000,000 won, middle income: 4,000,001~6,000,000 won, high income: >6,000,000 won from the basis of the income level of participants. ^(2)^ Obesity is defined according to BMI z-score ≥ 2, insulin resistance is defined by HOMA-IR ≥ 2.6, Metabolic syndrome is defined according to cardiovascular disease risk factor clustering (CVD-RFC) for children and adolescents. Defined as the presence of over 2 of following risk factors: Central Obesity (waist circumference) ≥ 90th percentile of adult cut-off, Triglycerides (mg/dL) ≥ 110, HDL cholesterol (mg/dL) < 40, BP (mmHg) SBP or DBP ≥ 90th percentile of adult cut-off, fasting plasma glucose (mg/dL) ≥ 100.

**Table 2:** Factor loading matrix for major dietary patterns in Korean children and adolescents.

	Dietary Patterns					
Food Groups	Fast Food & Soda	White Rice & Kimchi	Oil & Seasoned Vegetable			
White rice	−0.222	0.745	0.082			
Whole grain & others	−0.298	−0.064	0.001			
Snack & cereals	0.070	−0.482	−0.079			
Flour & rice cakes	−0.049	−0.281	0.040			
Instant ramen	0.298	0.182	−0.167		−0.75	
Noodles	−0.071	−0.260	0.188		−0.65	
Potatoes	−0.044	0.020	0.321		−0.55	
Sugars	−0.154	−0.264	0.519		−0.45	
Soups	−0.347	0.062	−0.121		−0.35	
Legumes	−0.268	0.014	0.096		−0.25	
Nuts	−0.263	−0.202	0.295		−0.15	
Vegetable & mushrooms	−0.369	0.076	0.259		0	
Kimchi	−0.147	0.424	0.139		0.15	
Fruit	−0.296	−0.160	−0.426		0.25	
Meat & fish	−0.209	0.028	0.080		0.35	
Eggs	0.027	0.440	0.041		0.45	
Seaweed	0.033	0.187	0.087		0.55	
Milk and dairy products	−0.413	−0.237	−0.417		0.65	
Vegetable oil	−0.151	0.310	0.647		0.75	
Carbonated beverages	0.583	−0.200	0.129			
Other drinks	−0.004	0.074	−0.080			
Seasonings	−0.325	0.144	0.449			
Fast food	0.702	−0.184	−0.079			
Processed food	−0.044	0.441	−0.102			
Fermented salty foods	−0.208	−0.132	0.122			

**Table 3:** Dietary intakes and metabolic risk factors according to tertile of dietary pattern for Korean children and adolescents ^(1),^*.

	Fast Food & Soda									*p* for Trend ^(2)^	White Rice & Kimchi									*p* for Trend	Oil & Seasoned Vegetable									*p* for Trend
	T1 (low)			T2			T3 (High)				T1 (Low)			T2			T3 (High)				T1 (Low)			T2			T3 (High)			
**Dietary intakes ^(3)^**																														
Energy (kcal)	2066.72	±	45.46 ^b^	2214.16	±	45.24 ^ab^	2256.44	±	45.47 ^a^	<0.0001	2264.08	±	44.76 ^b^	2252.70	±	44.77 ^ab^	2020.54	±	44.76 ^a^	<0.0001	2146.69	±	45.90	2233.89	±	45.62	2156.75	±	45.88	<0.0001
Carbohydrate (g)	294.37	±	3.45	292.13	±	3.41	284.07	±	3.44	<0.0001	281.73	±	3.40 ^c^	291.93	±	3.40 ^b^	296.92	±	3.43 ^a^	<0.0001	288.91	±	3.45	289.26	±	3.43	292.42	±	3.44	<0.0001
Protein (g)	86.68	±	1.14 ^a^	81.88	±	1.13 ^b^	84.99	±	1.14 ^ab^	<0.0001	84.72	±	1.14	85.10	±	1.14	83.73	±	1.15	<0.0001	84.29	±	1.14	85.35	±	1.14	83.91	±	1.14	<0.0001
Fat (g)	71.16	±	1.24 ^b^	73.41	±	1.23 ^ab^	75.46	±	1.24 ^a^	<0.0001	77.10	±	1.22 ^b^	72.46	±	1.22 ^a^	70.46	±	1.23 ^a^	<0.0001	74.37	±	1.24	73.19	±	1.23	72.47	±	1.24	<0.0001
C:P:F ratio	54.4:16.0:29.6			54.2:15.2:30.6			52.7:15.8:31.5				52.2:15.7:32.1			54.1:15.8:30.2			55.1:15.5:29.4				53.4:15.6:31.0			53.6:15.8:30.5			54.2:15.6:30.2			
**Anthropometric**																														
Height (cm)	151.70	±	0.62	151.92	±	0.61	151.57	±	0.61	<0.0001	152.29	±	0.61	151.34	±	0.61	151.56	±	0.62	<0.0001	152.57	±	0.61	151.50	±	0.61	151.11	±	0.61	<0.0001
Weight (kg)	61.06	±	1.32	62.56	±	1.30	62.86	±	1.31	<0.0001	62.45	±	1.31	62.23	±	1.31	61.79	±	1.32	<0.0001	63.80	±	1.31	62.02	±	1.30	60.65	±	1.31	<0.0001
WC (cm)	82.23	±	1.11	84.56	±	1.10	85.26	±	1.11	<0.0001	83.20	±	1.11	84.83	±	1.11	84.02	±	1.12	<0.0001	84.58	±	1.11	84.61	±	1.11	82.86	±	1.11	0.013
HC (cm)	92.75	±	0.90	94.39	±	0.89	95.06	±	0.90	N/S	94.18	±	0.90	94.31	±	0.90	93.70	±	0.91	<0.0001	94.41	±	0.90	94.25	±	0.90	93.53	±	0.90	<0.0001
WHR	0.88	±	0.01	0.89	±	0.01	0.89	±	0.01	<0.0001	0.87	±	0.01 ^a^	0.90	±	0.01^b^	0.89	±	0.01^ab^	<0.0001	0.89	±	0.01	0.89	±	0.01	0.88	±	0.01	<0.0001
BMI (kg/m^2^)	25.81	±	0.44	26.52	±	0.43	26.84	±	0.44	<0.0001	26.27	±	0.44	26.59	±	0.44	26.31	±	0.44	<0.0001	26.75	±	0.44	26.45	±	0.44	25.97	±	0.44	0.981
BMI z-score	1.99	±	0.13	2.22	±	0.13	2.30	±	0.13	<0.0001	2.14	±	0.13	2.25	±	0.13	2.13	±	0.13	<0.0001	2.26	±	0.13	2.20	±	0.13	2.06	±	0.13	<0.0001
SBP (mmHg)	115.54	±	1.37	116.91	±	1.35	118.32	±	1.36	N/S	117.16	±	1.36	116.58	±	1.36	117.03	±	1.37	N/S	117.40	±	1.36	117.48	±	1.35	115.88	±	1.36	N/S
DBP (mmHg)	67.09	±	0.85	67.63	±	0.84	68.02	±	0.85	0.902	67.19	±	0.85	67.53	±	0.84	68.02	±	0.85	<0.0001	67.90	±	0.85	67.48	±	0.84	67.48	±	0.84	<0.0001
**Biochemistry**																														
Glucose (mg/dL)	88.68	±	0.74	89.47	±	0.73	90.27	±	0.73	N/S	89.52	±	0.73	89.57	±	0.73	89.33	±	0.74	<0.0001	89.69	±	0.73	90.02	±	0.73	88.72	±	0.73	<0.0001
Insulin (uU/mL)	18.13	±	1.11	19.65	±	1.10	20.67	±	1.11	<0.0001	19.40	±	1.11	19.93	±	1.10	19.11	±	1.12	<0.0001	20.57	±	1.11	19.84	±	1.10	18.03	±	1.10	<0.0001
HOMA-IR	3.98	±	0.28	4.33	±	0.27	4.66	±	0.28	<0.0001	4.35	±	0.27	4.42	±	0.27	4.21	±	0.28	<0.0001	4.54	±	0.27	4.49	±	0.27	3.94	±	0.27	<0.0001
Triglyceride (mg/dL)	99.21	±	4.39	105.18	±	4.34	111.60	±	4.37	<0.0001	101.83	±	4.32^ab^	97.91	±	4.32^b^	116.26	±	4.37^a^	<0.0001	110.08	±	4.37	102.69	±	4.35	103.22	±	4.37	<0.0001
HDL cholesterol (mg/dL)	52.18	±	1.04	52.15	±	1.03	52.00	±	1.03	<0.0001	51.29	±	1.03	52.66	±	1.03	52.37	±	1.04	<0.0001	51.64	±	1.03	52.74	±	1.03	51.94	±	1.03	<0.0001
LDL cholesterol (mg/dL)	108.70	±	2.11	109.36	±	2.09	110.60	±	2.11	0.902	109.91	±	2.10	110.09	±	2.10	108.66	±	2.12	0.714	110.37	±	2.10	111.07	±	2.09	107.22	±	2.10	<0.0001

Abbreviation: T, tertile; C:P:F ratio, ratio of carbohydrate to protein to fate; SBP, systolic blood pressure; DBP, diastolic blood pressure; WC, waist circumference; HC, hip circumference; WHR, waist–hip ratio; BMI, body mass index; HOMA-IR, homeostatic model assessment for insulin resistance. ^(1)^ Values are LS mean ± SE adjusted for age, sex and energy intake from general linear model (GLM) for quantitative variables. ^(2)^ Trend analysis for general linear model using the median values for tertiles of dietary pattern. ^(3)^ Reported means for nutrients intake are adjusted for age, sex, and energy intake. * Letters ^(a,b,c)^ with different superscripts in the same row indicate significantly different values at *p* < 0.05 according to Tukey post hoc test.

**Table 4:** Associations between dietary patterns and metabolic risk factors in Korean children and adolescents.

	Fast Food & Soda		White Rice & Kimchi		Oil & Seasoned Vegetable	
	β	*p*-Value	β	*p*-Value	β	*p*-Value
Height (cm)	0.00	<0.0001	−0.32	<0.0001	−0.73	<0.0001
Weight (kg)	1.00	<0.0001	−0.24	<0.0001	−1.57	<0.0001
SBP (mmHg)	1.31	<0.0001	−0.17	<0.0001	−0.76	<0.0001
DBP (mmHg)	0.53	0.157	0.46	0.166	−0.26	0.189
WC (cm)	1.55	<0.0001	0.44	<0.0001	−0.86	<0.0001
HC (cm)	1.18	<0.0001	−0.23	<0.0001	−0.44	<0.0001
WHR (%)	0.01	<0.0001	0.01	<0.0001	−0.00	<0.0001
BMI (kg/m^2^)	0.53	<0.0001	0.03	<0.0001	−0.39	<0.0001
BMI z score	0.16	0.001	0.00	0.004	−0.10	0.001
Glucose (mg/dL)	0.84	0.425	−0.06	0.747	−0.49	0.631
Insulin (uU/mL)	1.25	<0.0001	−0.16	<0.0001	−1.27	<0.0001
HOMA-IR	0.34	<0.0001	−0.07	0.000	−0.30	<0.0001
Triglyceride (mg/dL)	6.03	0.112	7.03	0.070	−3.50	0.247
HDL cholesterol (mg/dL)	−0.03	0.001	0.58	0.001	0.17	0.001
LDL cholesterol (mg/dL)	1.00	0.938	−0.59	0.959	−1.58	0.872

Abbreviations: SBP, systolic blood pressure; DBP, diastolic blood pressure; WC, waist circumference; HC, hip circumference; WHR, waist–hip ratio; BMI, body mass index; BMI z-score, body mass index z-score; HOMA-IR, homeostatic model assessment for insulin resistance. Multiple linear regression analysis between tertiles of dietary pattern score and metabolic risk factors adjusted by age, sex and energy intake and house income, and parental education level. Values are expressed as β coefficients.

**Table 5:** Associations (OR and 95% CI) between tertiles of dietary patterns and obesity, insulin resistance, and metabolic syndrome in Korean children and adolescents.

	Fast Food & Soda			*p* for Trend	White Rice & Kimchi			*p* for Trend	Oil & Seasoned Vegetable			*p* for Trend
	OR (95% CI)				OR (95% CI)				OR (95% CI)			
	T1	T2	T3		T1	T2	T3		T1	T2	T3	
BMI z score ≥ 2												
Model I ^(1)^	1.00	1.36	1.67	0.043	1.00	1.31	0.76	0.255	1.00	0.85	0.85	0.527
		(0.836, 2.220)	(1.013, 2.756)			(0.786, 2.194)	(0.466, 1.234)			(0.516, 1.401)	(0.516, 1.401)	
Model II ^(2)^	1.00	1.25	1.49	0.654	1.00	1.31	0.83	0.099	1.00	0.79	0.793	0.858
		(0.762, 2.058)	(0.890, 2.482)			(0.781, 2.199)	(0.501, 1.364)			(0.473, 1.309)	(0.476, 1.323)	
HOMA-IR ≥ 2.6												
Model I	1.00	1.84	2.41	0.000	1.00	1.45	1.26	0.362	1.00	1.29	0.80	0.362
		(1.113, 3.032)	(1.430, 4.068)			(0.870, 2.425)	(0.761, 2.083)			(0.759, 2.181)	(0.483, 1.314)	
Model II	1.00	1.73	2.11	0.008	1.00	1.47	1.41	0.114	1.00	1.16	0.67	0.206
		(1.032, 2.905)	(1.227, 3.638)			(0.865, 2.484)	(0.831, 2.403)			(0.671, 1.997)	(0.396, 1.132)	
Metabolic Syndrome ^(3)^												
Model I	1.00	1.63	1.79	0.016	1.00	1.03	1.09	0.717	1.00	1.09	0.94	0.809
		(1.016, 2.609)	(1.109, 2.871)			(0.642, 1.650)	(0.680, 1.753)			(0.679, 1.757)	(0.589, 1.512)	
Model II	1.00	1.57	1.63	0.039	1.00	0.97	1.18	0.578	1.00	1.05	0.91	0.578
		(0.967, 2.556)	(0.993, 2.672)			(0.600, 1.575)	(0.716, 1.928)			(0.641, 1.707)	(0.559, 1.494)	

Abbreviations: OR, odds ratio; CI, confidence interval; T, tertile; BMI, body mass index; WC, waist circumference; SBP, systolic blood pressure; DBP, diastolic blood pressure. ^(1)^ Model I was not adjusted results of logistic regression analysis between obesity, insulin resistance, and metabolic syndrome and tertiles of dietary pattern. ^(2)^ Model II was the results of logistic regression analysis adjusted for age, sex, and energy intake, household income, and parental education level between obesity, insulin resistance, and metabolic syndrome and tertiles of dietary pattern. ^(3)^ Metabolic syndrome is defined according to cardiovascular disease risk factor clustering (CVD-RFC) for children and adolescents. Defined as the presence of over 2 of following risk factors: Obesity (waist circumference) ≥ 90th percentile of adult cut-off, Triglycerides (mg/dL) ≥ 110, HDL cholesterol (mg/dL) < 40, BP (mmHg) SBP or DBP ≥ 90th percentile of adult cut-off, fasting plasma glucose (mg/dL) ≥ 100.

## Supplementary Materials

The following are available online at https://www.mdpi.com/article/10.3390/nu13041153/s1, Table S1: Food grouping for Korean children and adolescents in subject.
