# Supplementary material for: Association of Dietary Patterns with Weight Status and Metabolic Risk Factors among Children and Adolescents
Source: Nutrients. 2021 Mar 31;13(4):1153. doi: 10.3390/nu13041153 (PMC8065894; doi:10.3390/nu13041153)
Supplement: Supplementary file 1 [file nutrients-13-01153-s001.pdf]

Table S1. Food groupings for Korean children and adolescents

	Foods or food groups	Food items
1	White rice	Steamed rice, rice congee
2	Whole grain & others	Grains, acorn jelly (called dotori-muk), corn, Korean multigrain shakes (misugaru latte)
3	Snack & cereals	Snack, cookies, cake, pudding, popcorn, chocolate, ice cream, candy, sweet jelly of red beans cereals
4	Flour & rice cakes	Bread, dumpling, tortilla, sandwiches, soup, pie, rice cake, tteokbokki (spicy rice cake)
5	Instant ramen	Instant ramen
6	Noodles	Noodle in black bean sauce (jajangmyeon), pasta, Korean knife cut noodle soup (kalguksu)
7	Potatoes	Sweet potato, potato, potato salad, starch from potatoes
8	Sugars	Sugar, jam, syrup, honey, starch syrup
9	Soup	Korean soybean paste soup (doenjang guk) , Kimchi soup, squid soup (ojingeo-guk) , Korean seaweed soup (miyeok guk) , spicy beef soup with vegetables (yukgaejang)
10	Legumes	Beans Soy bean paste, tofu, fried tofu, tofu residue
11	Nuts	Sesame, perilla, walnut, almond, pine nut, sunflower seed
12	Vegetable & mushrooms	Cucumber, paprika, lettuce, spinach, cabbage, radish, carrot, onion, broccoli, mushroom
13	Kimchi	Kimchi, kkakdugi (cubed radish kimchi)
14	Fruit	Fruit, canned fruit
15	Meat & fish	Pork, pork belly, pork intestines, beef, beef ribs, beef intestines, chicken, duck meat, turkey, Sushi, largehead hairtail, eel, larimichthys polyactis , alaska pollock, mackerel, tuna, salmon
16	Eggs	Eggs
17	Seaweed	Seaweed, kelp
18	Milk and dairy products	Milk, yogurt, cheese
19	Vegetable oil	Sesame oil, perilla oil, soybean oil
20	Carbonated beverages	Coke, sprite
21	Other drinks	Sweet drinks, fruit juice, Green tea, red ginseng tea, soy bean milk
22	Seasonings	Salt, black pepper, soy sauce, dressing, curry, red pepper paste, ketchup, mayonnaise
23	Fast food	Burger, French fries, fried chicken, fried chicken with sauce, pizza
24	Processed food	Ham, sausage, bacon, pork cutlet, fish cutlet, fish cake
25	Fermented salty foods	Fermented seafood, pickled vegetable

Note: General food groupings were based on similarities between foods and nutrient composition.
